# Supplementary material for: Cohesin Facilitates Nucleosome Invasion by Transcription Factors
Source: bioRxiv. 2025 Dec 10:2025.12.08.693039. Preprint. [Version 1] doi: 10.64898/2025.12.08.693039 (PMC12712941; doi:10.64898/2025.12.08.693039)
Supplement: 1 [file NIHPP2025.12.08.693039v1-supplement-1.pdf]

(which was not certified by peer review) is the author/funder, who has granted bioRxiv a license to display the preprint in perpetuity. It is m
available under aCC-BY 4.0 International license.

Figure S1

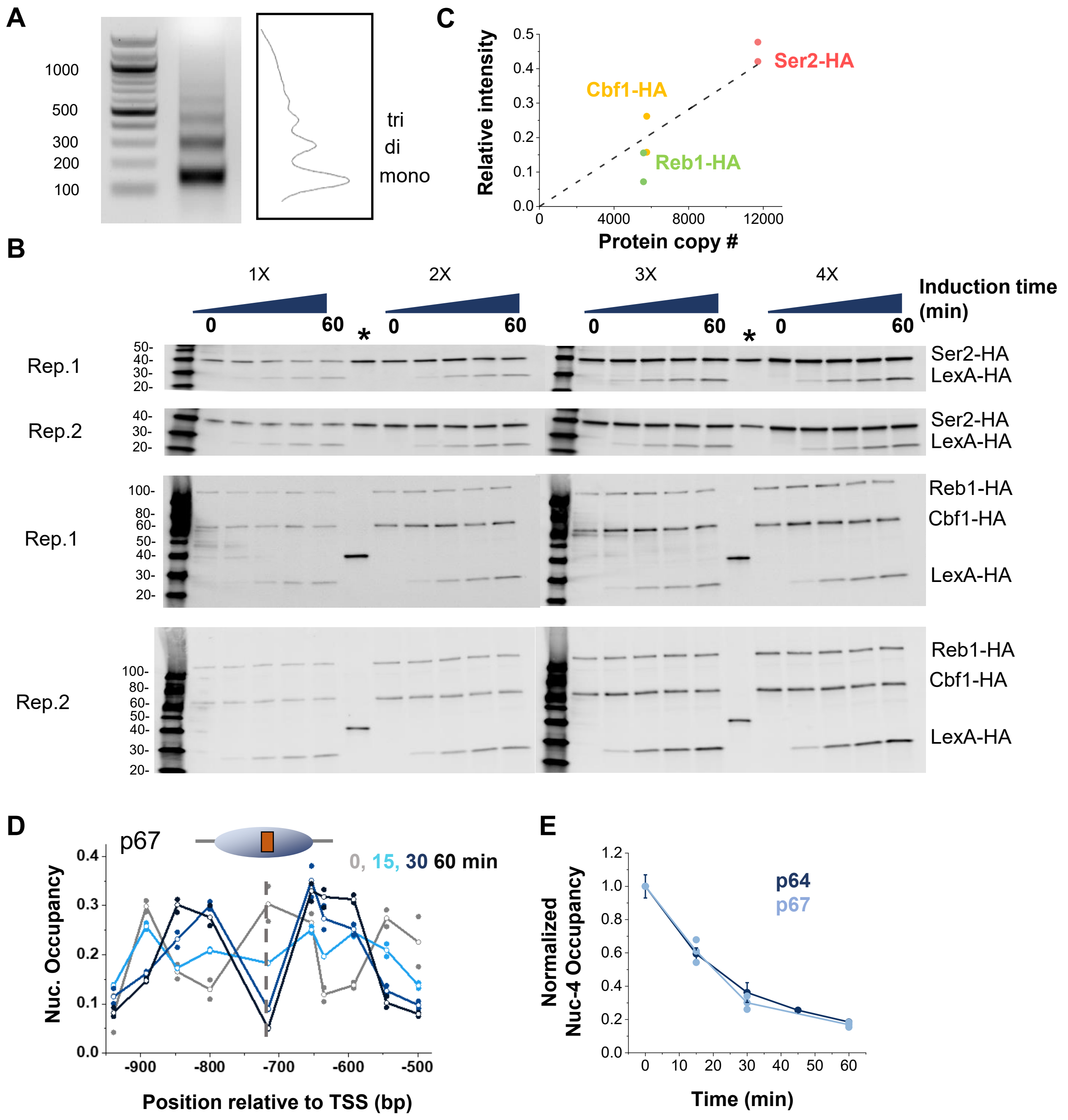

Figure S1. Additional LexA invasion and quantification data, related to Figure 1.

A) Representative MNase gel and the corresponding ImageJ intensity plot showing mono-, di-, and tri-nucleosome bands. Mononucleosome bands were gel extracted for nucleosome mapping by qPCR.

B) Western blots (two biological replicates for each time point and strain) used for quantifying LexA-HA levels in Figure 1H. Four different cell lyaste concentrations were loaded ensure that each time point fell within the linear detection range of the assay. The asterisks mark the gel lanes containing internal HA loading control (see Methods). Molecular weight markers indicated in first lane.

C) Band intensity vs the known copy number of Cbf1, Reb1, and Ser2 (from SGD). The linear fit (gray curve) was used to estimate the copy number of LexA.

D) Time course of LexA invasion with a slightly shifted LexO position in nucleosome –4. The original dyad position of LexO in Figure 1D is p64, here shifted by 3 bp to p67.

E) Quantification of nucleosome depletion during LexA induction over the LexO site at p64 and p67.

(which was not certified by peer review) is the author/funder, who has granted bioRxiv a license to display the preprint in perpetuity. It is m
available under aCC-BY 4.0 International license.

Figure S2

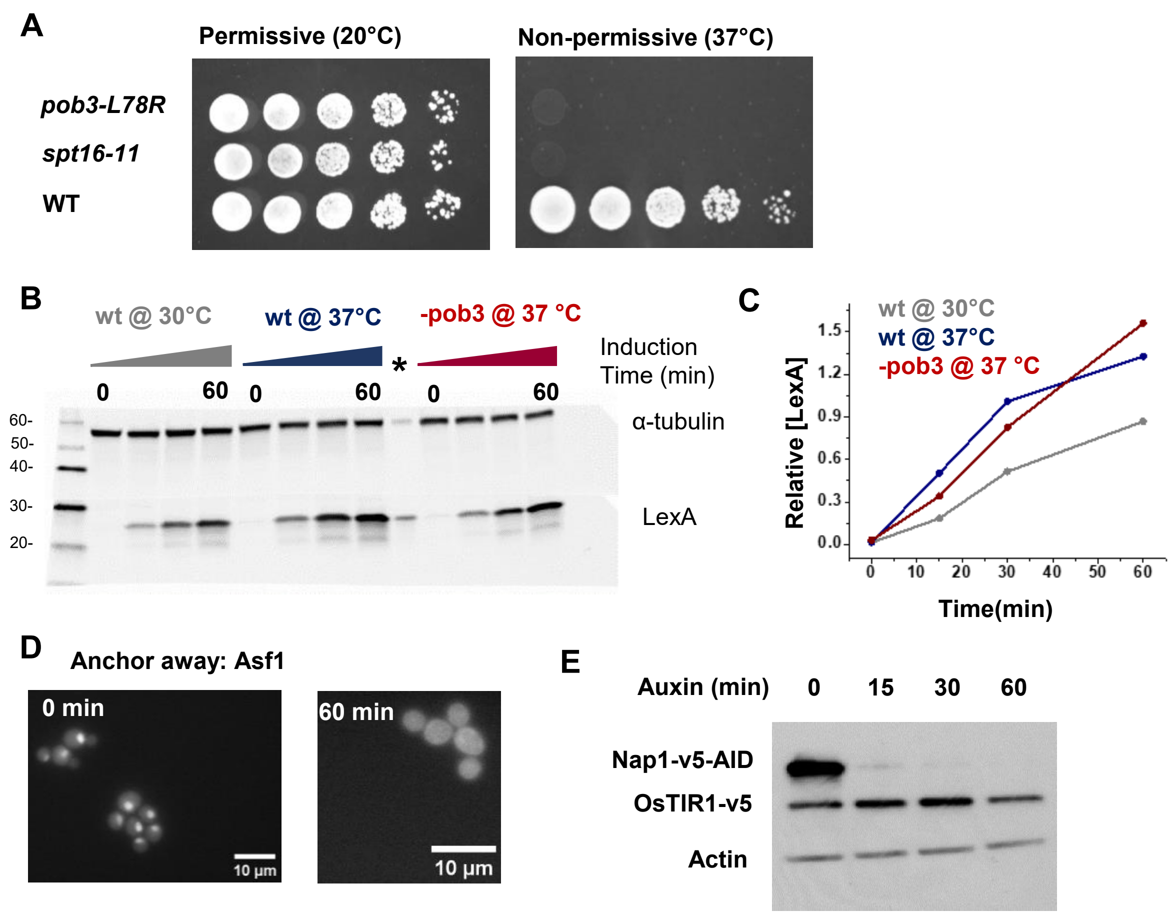

Figure S2. Histone chaperone depletion and LexA induction, related to Figure 3.
A) Spotting-assay for WT yeast and two temperature-sensitive FACT mutants grown at permissive (20°C) and nonpermissive (37°C) temperatures.
B) Western blot analysis of LexA induction in WT and FACT mutant strains. Molecular weights are indicated in the first lane.
C) Quantification of LexA levels in the Western blot in B.
D) Fluorescence images showing anchor-away of Asf1-FRB-GFP following rapamycin treatment.
E) Western blot confirming auxin-induced degradation of Nap1.

(which was not certified by peer review) is the author/funder, who has granted bioRxiv a license to display the preprint in perpetuity. It is m
available under aCC-BY 4.0 International license.

Figure S3

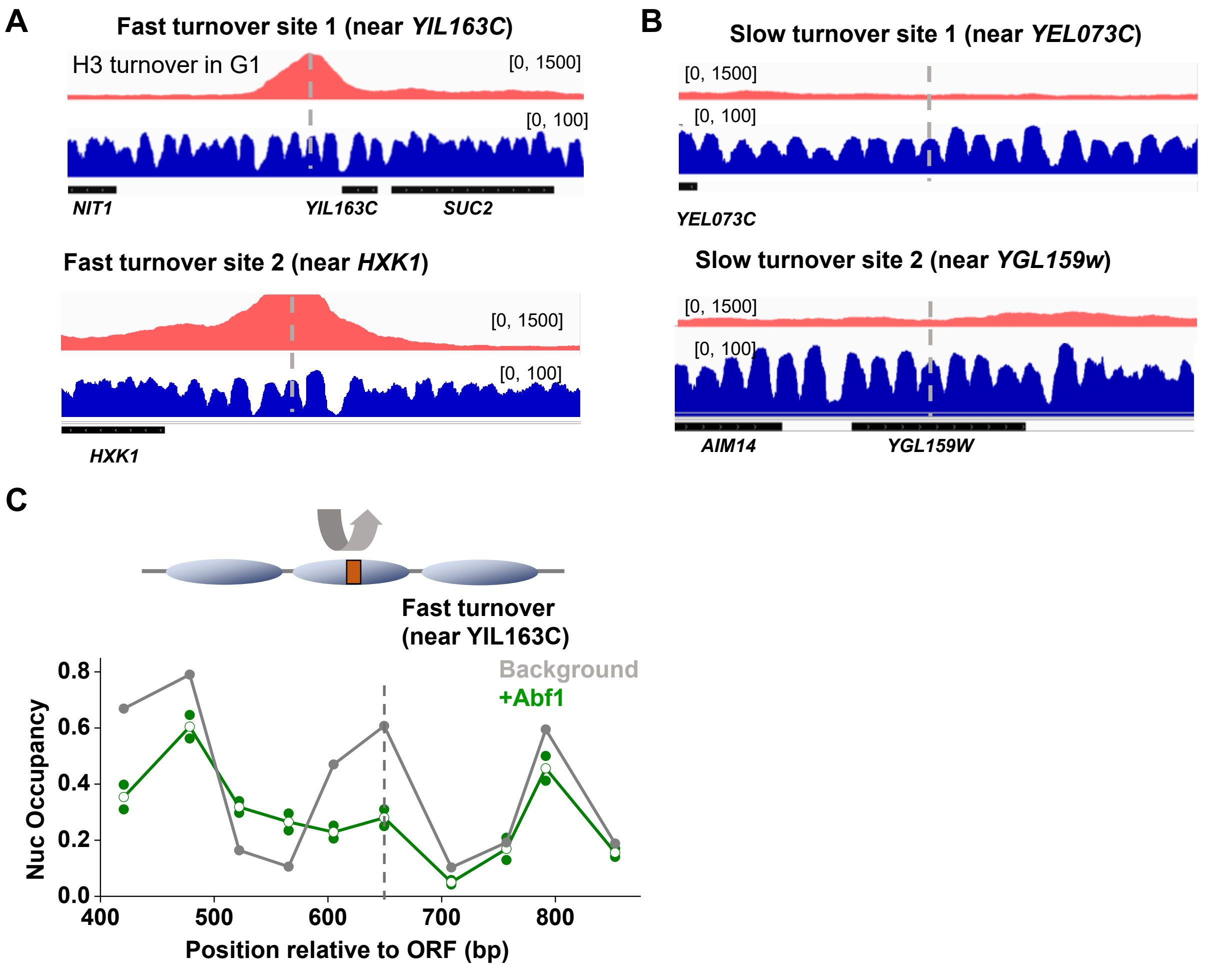

Figure S3. Histone turnover data and Abf1 invasion, related to Figure 4.
A) IgV tracks showing histone turnover (red) and nucleosome occupancy (blue) near the two fast turnover sites used in Figure 4B. The histone turnover data are from Kassem et al., and the Mnase-seq data are generated by our own lab. The dashed lines mark the locations of LexO.
B) Same as in A except for the two slow turnover sites used in Figure 4C.
C) Nucleosome occupany near an Abf1 motif engineered into a fast turnover nucleosome (same site as the left panel in Figure 4B). Nucleosome in this case is displaced by the endogenous Abf1.

(which was not certified by peer review) is the author/funder, who has granted bioRxiv a license to display the preprint in perpetuity. It is m
available under aCC-BY 4.0 International license.

Figure S4

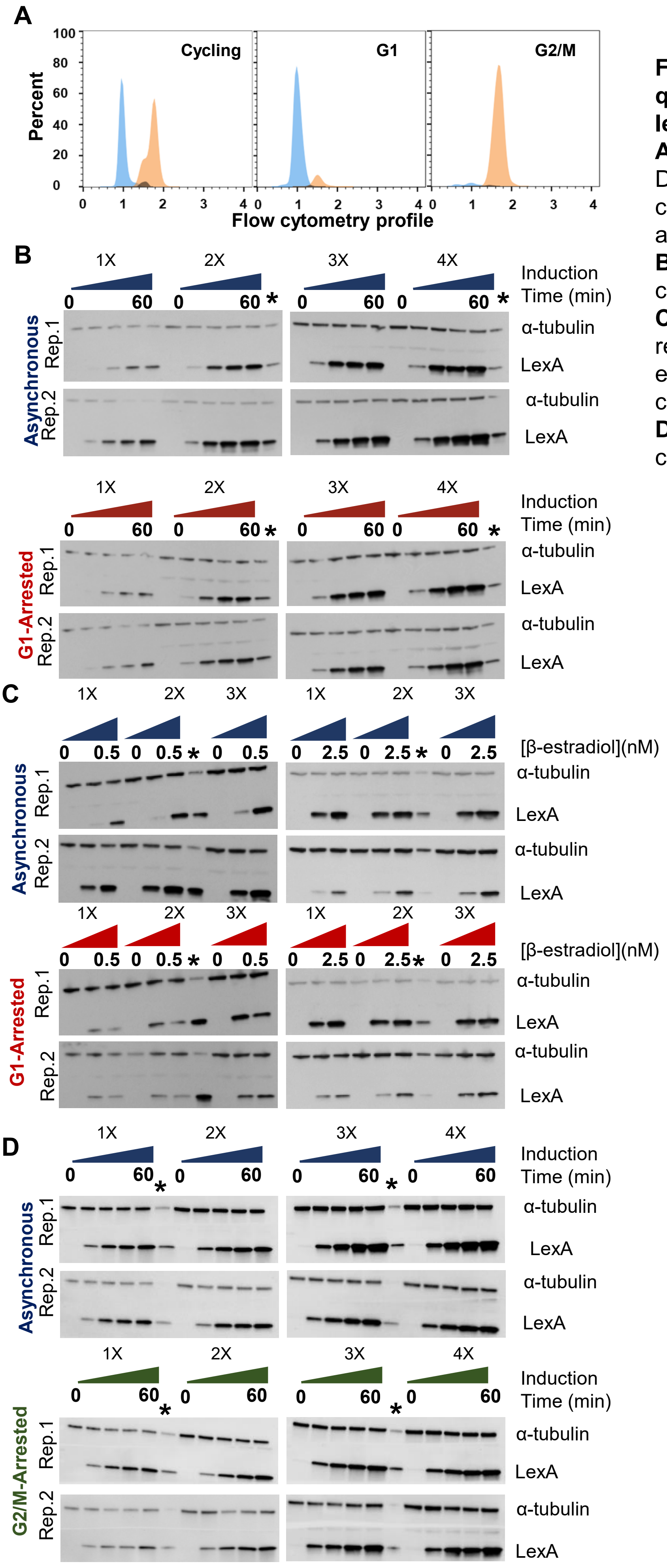

Figure S4. Cell cycle arrest and quantification of LexA induction levels, relate to Figure 5.
A) Flow cytometric analysis of the DNA content in cycling cells (left), cells arrest in G1 by α factor (middle) and in G2/M by nocodazole (right).
B) Western blot of LexA induction in cycling vs G1-arrested cells.
C) Western blot of LexA induction in response to variable amount of β-estradiol in cycling vs G1-arrested cells.
D) Western blot of LexA induction in cycling vs G2/M-arrested cells.

(which was not certified by peer review) is the author/funder, who has granted bioRxiv a license to display the preprint in perpetuity. It is m
available under aCC-BY 4.0 International license.

Figure S5

A

Category	Factors
Histone modifying enzymes	Ada2, Hif1, Hsf3, Rtt109, Set3
Topoisomerase	Top2, Top3
Cohesin-associated	Smc1, Smc3, Mcd1, Pds5

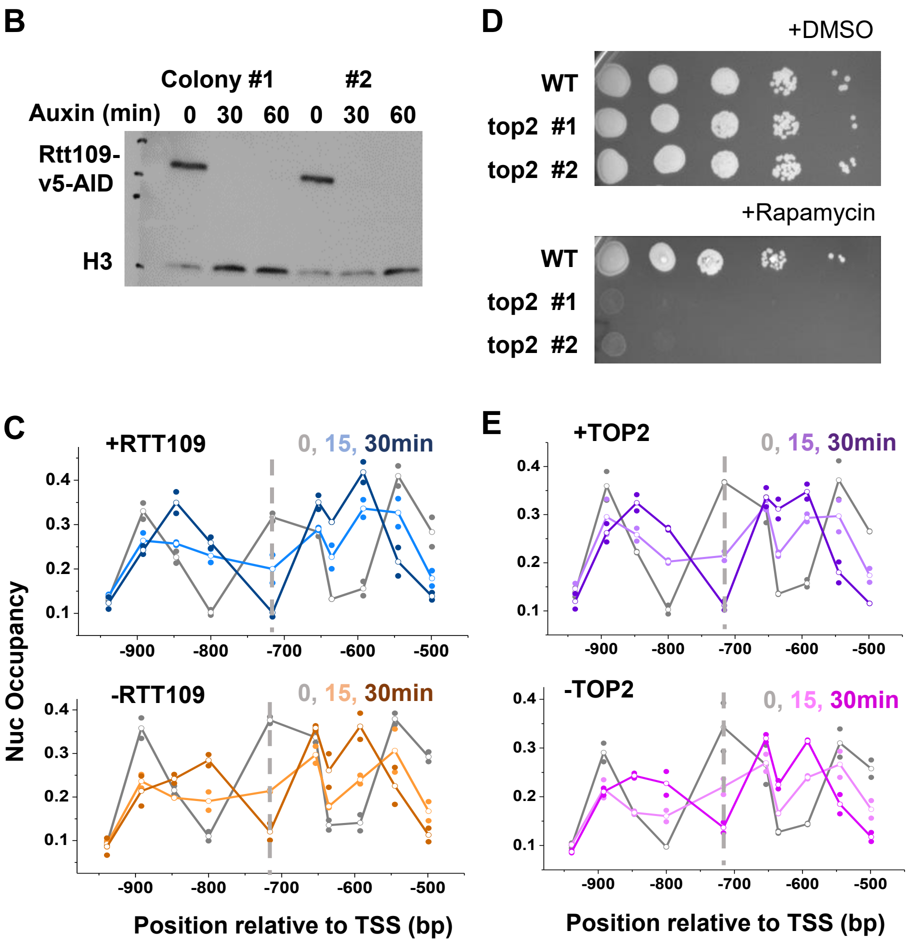

Figure S5. Nucleosome invasion by LexA is not affected by Top2 and Rtt109, related to Figure 6.
A) List of chromatin-related factors that are transcriptionally repressed in G1.
B) Western blot confirming auxin-induced degradation of Rtt109.
C) Time course of LexA invasion into nucleosome –4 in cells with or without Rtt109.
D) Spotting assay confirming the lethality of cells with Top2 anchor away.
E) Time course of LexA invasion into nucleosome –4 in cells with or without Top2.

(which was not certified by peer review) is the author/funder, who has granted bioRxiv a license to display the preprint in perpetuity. It is m
available under aCC-BY 4.0 International license.

Figure S6

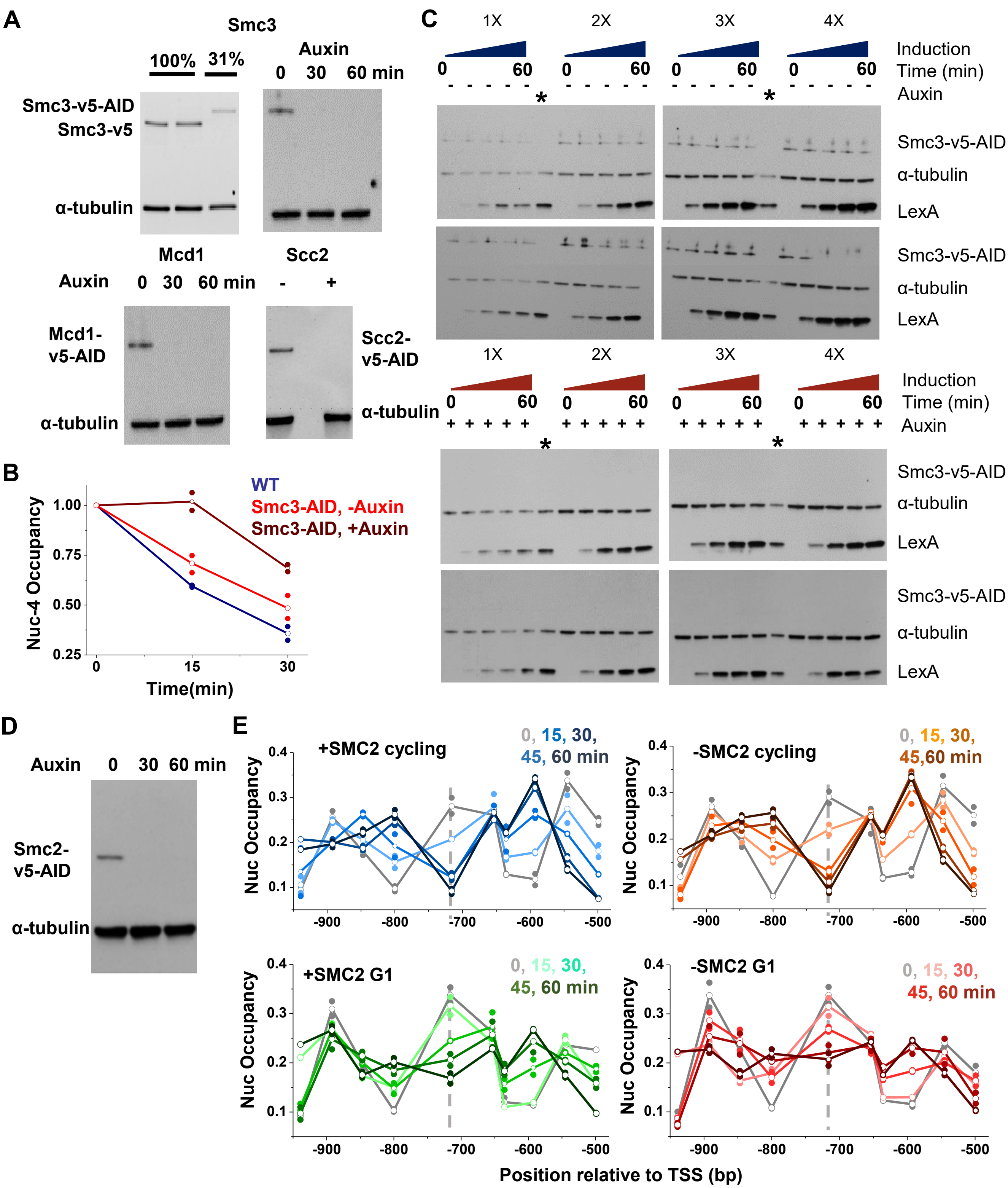

Figure S6. LexA expression and nucleosome invasion in the absence of cohesin or condensin, related to Figure 6.

A) Western blots confirming auxin-induced degradation of Smc3, Mcd1, and Scc2. For Smc3, we also compared the WT level (Smc3-v5) to Smc3-v5-AID with no Auxin (upper left panel), which shows significant basal degradation.

B) Nucleosome occupancy change over the LexO site upon LexA induction in WT and Smc3-AID ±auxin conditions.

C) Western blot measuring LexA induction level in the presence and absence of Smc3. **D)** Western blot confirming auxin-induced degradation of Smc2.

E) Time course of LexA invasion into nucleosome –4 in Smc2-AID strain ±auxin in cycling vs G1-arrested cells.

(which was not certified by peer review) is the author/funder, who has granted bioRxiv a license to display the preprint in perpetuity. It is m
available under aCC-BY 4.0 International license.

Figure S7

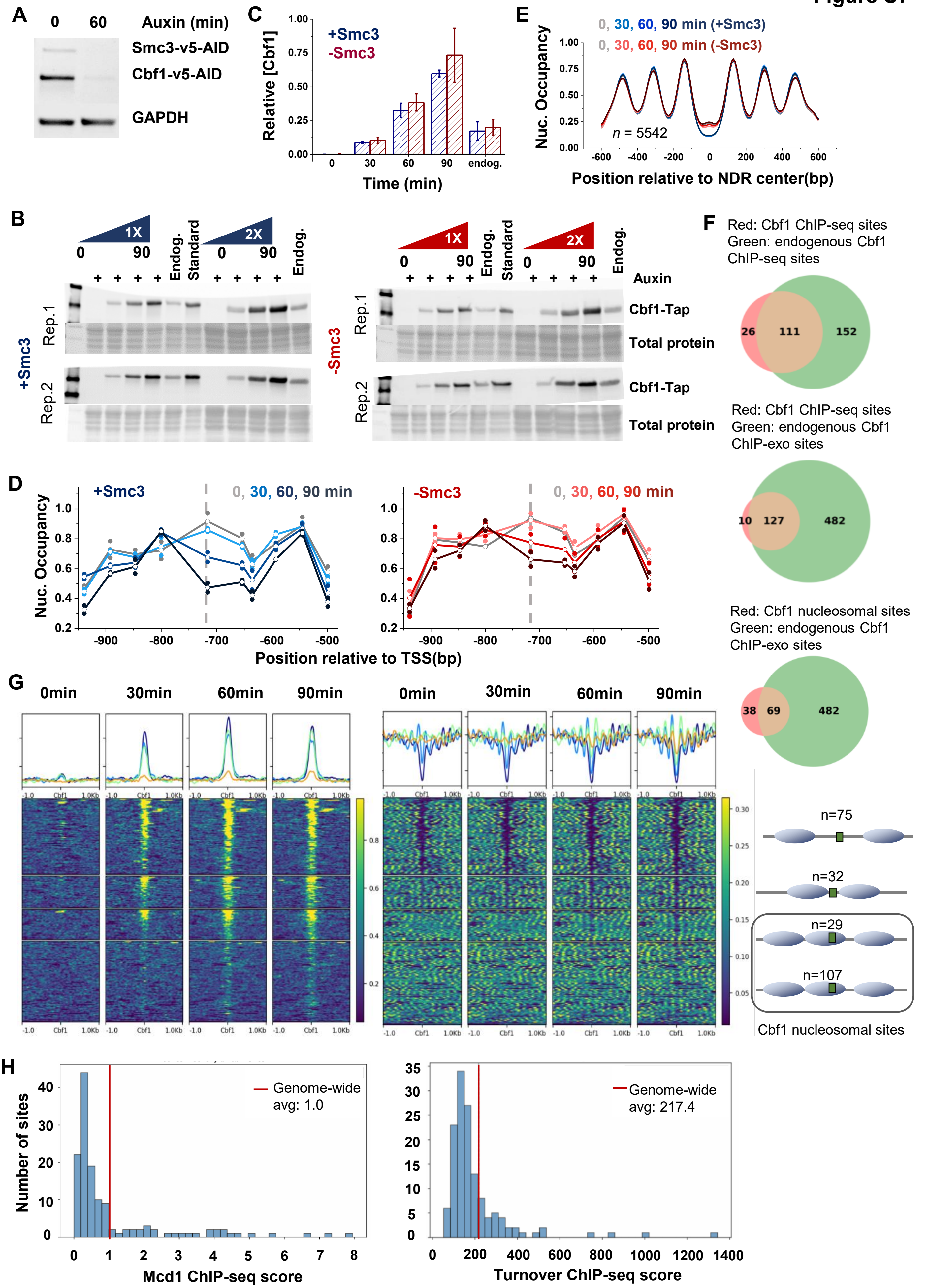

(which was not certified by peer review) is the author/funder, who has granted bioRxiv a license to display the preprint in perpetuity. It is m
available under aCC-BY 4.0 International license.

Figure S7. Cbf1 binding and nucleosome invasion in the presence or absence of Smc3, related to Figure 7.

A) Western blot confirming auxin-induced degradation of Smc3 and Cbf1.

B) Western blot analysis of Cbf1 induction levels in the presence or absence of Smc3. Total protein levels were used as loading controls (see Methods).

C) Quantification of Cbf1 expression levels following induction in WT versus Smc3-depleted cells, and comparison with endogenous Cbf1 level.

D) Time course of Cbf1 invasion into nucleosome –4 in the presence or absence of Smc3.

E) Average nucleosome occupancy profile near genome-wide NDRs, aligned at NDR centers, in ± Smc3 cells at all time points of Cbf1 induction.

F) Overlap of Cbf1 binding sites detected in this study with previously published datasets.

G) ChIP-seq (left) and MNase-seq (right) over different classes of Cbf1 binding sites. From top to bottom: 1) sites in wide NDRs, 2) sites in narrow NDRs or nucleosome edges, 3) sites embedded within nucleosomes with strong Cbf1 ChIP-seq signals, and 4) sites within nucleosomes with weak Cbf1 ChIP-seq signals. The last two groups are combined as nucleosomal sites.

H) Mcd1 ChIP-seq signals (left) and H3 turnover signals (right) at nucleosome-embedded Cbf1 sites. Genome-wide averages are marked by vertical bars and used as thresholds to group sites into high- versus low-signal categories.
